# Household Compositions and Substance Use among Young Adults in the U.S.

**DOI:** 10.3390/ijerph21081067

**Published:** 2024-08-14

**Authors:** Beth Han, Naomi Tomoyasu, Emily B. Einstein, Christopher M. Jones, Wilson M. Compton

**Affiliations:** 1 National Institute on Drug Abuse, Bethesda, MD 20892, USA; emily.einstein@nih.gov (E.B.E.); wcompton@nida.nih.gov (W.M.C.); 2 Center for Behavioral Health Statistics and Quality, Substance Abuse and Mental Health Services Administration, Rockville, MD 20852, USA; naomi.tomoyasu@samhsa.hhs.gov; 3 Center for Substance Abuse Prevention, Substance Abuse and Mental Health Services Administration, Rockville, MD 20852, USA; christopher.jones@samhsa.hhs.gov

**Keywords:** binge alcohol use, tobacco use, drug use, residing with own children, residing with own children and a spouse or partner, residing solely with unrelated individuals, the National Survey on Drug Use and Health

## Abstract

Background: Adults aged 21–29 have the highest past-month prevalence of tobacco, alcohol, and illicit drug use in the U.S. Currently, young adults often delay traditional adulthood milestones (e.g., marriage and childbearing), which may impact their household composition and substance use. Methods: We examined how the past-month prevalence of eight mutually exclusive substance use outcomes varied by household composition among young adults using the 2016–2019 National Surveys on Drug Use and Health (NSDUH) data. Bivariable and multivariable multinomial logistic regression analyses were applied. Results: Among young adults residing with their children, the most common household composition was residing with children and a spouse/partner (16.6%, 95% CI = 16.5–16.8%). Among those residing without children, common household compositions included residing with parents (22.8%, 95% CI = 22.2–23.4%) and residing only with a spouse/partner (17.9%, 95% CI = 17.6–18.3%). Past-month prevalence of binge alcohol, tobacco, and illicit drug use varied by household composition. Residing only with children and a spouse/partner was associated with a low prevalence of most examined substance use patterns. Across household compositions, those residing solely with unrelated individuals had the highest adjusted prevalence of tobacco, drug, and binge alcohol use (13.8%, 95% CI = 12.5–15.1%). Conclusions: The prevalence of substance use patterns among U.S. young adults varies by household composition. Those residing solely with unrelated individuals had the highest prevalence of tobacco, binge alcohol, and drug use. The presence of a young adult’s own children and a spouse/partner is associated with a lower prevalence of most examined substance use patterns. As household compositions continue to diversify, targeted substance use prevention/treatment strategies may be needed.

## 1. Introduction

Although life course transitions, such as employment, marriage, and childbearing, are associated with reduced risk of substance use [1,2,3,4], U.S. young adults have been achieving these key milestones of adulthood at a later age than their parents [5]. For example, the mean age of U.S. mothers at first birth was 27.3 years in 2021, a record high for the nation [6]. Moreover, the percentage of U.S. households in which adults reside with their child(ren), a household composition type associated with reduced opioid misuse [7], has been decreasing, and the decline is projected to continue [8].

Despite demographic changes among young adults [1,2,3,4,6,8], detailed types and distributions of household composition among 21–29-year-olds in the U.S. have not been recently assessed. Furthermore, none of the existing research has examined associations between household compositions and use patterns of specific substances (i.e., tobacco, binge alcohol, and drugs) among young adults in the U.S. Yet, young adults (i.e., those aged 21–29) have the highest past-month prevalence of tobacco, alcohol, and illicit drug use across all age groups in the U.S. [1,9], and polysubstance use is common among this population [10].

Previous studies have reported differences in associations with illicit drug and alcohol use among adolescents residing with one parent vs. two parents [11,12,13,14]. For parents residing with their child(ren), their substance use has been associated with an increased risk of substance use among their offspring [15,16]. However, these findings may not apply to young adults who are at the stage of establishing their own households and may not yet be parents. Notably, many young adults do not live with any relatives. Over half of adults aged 18–24 lived alone or lived with unrelated roommates in 2017 [17].

In response, we examined U.S. nationally representative data to understand whether and how the prevalence of tobacco, binge alcohol, and drug use varied by household composition among young adults. As household compositions continue to diversify, our results may help inform substance use prevention and intervention strategies for U.S. young adults directly at the household level and help prevent and reduce their substance use.

## 2. Methods

### 2.1. Data Sources

Data were from individuals aged 21–29 who participated in the 2016–2019 National Surveys on Drug Use and Health (NSDUH), providing nationally representative data on tobacco, alcohol, and drug use among U.S. civilian noninstitutionalized adult populations [1,2,6,18]. The NSDUH data collection protocol was approved by the Institutional Review Board (IRB) at the Research Triangle Institute International. NSDUH employed a stratified multistage area probability sample. After dwelling unit selections were made, an interviewer visited each selected dwelling unit to establish a roster of all people residing in the dwelling unit and then 0–2 people were selected from the roster information for in-person interviews, as described below [1,2,6,18].

The 2016–2019 NSDUH were collected by interviewers in personal visits to households and noninstitutional group quarters. Each participant provided verbal informed consent. Audio computer-assisted self-administered interviewing was used, providing respondents with a private confidential way to record answers. Race and ethnicity were determined according to NSDUH respondents’ self-classification of racial and ethnic origin and identification based on classifications developed by the US Census Bureau. For the 2016–2019 NSDUH (pooled data for sufficient sample size), the weighted household screening response rate was 74.2% and the weighted interview response rate was 66.8%. Due to potential data disclosure risk, detailed household composition variables are only available in restricted NSDUH data files and are unavailable in NSDUH public use files. Thus, this study analyzed restricted deidentified NSDUH data [19] and was deemed exempt from review by the U.S. National Institutes of Health IRB. We followed the STROBE reporting guideline for observational (cross-sectional) studies.

### 2.2. Measures

**Figure 1:**
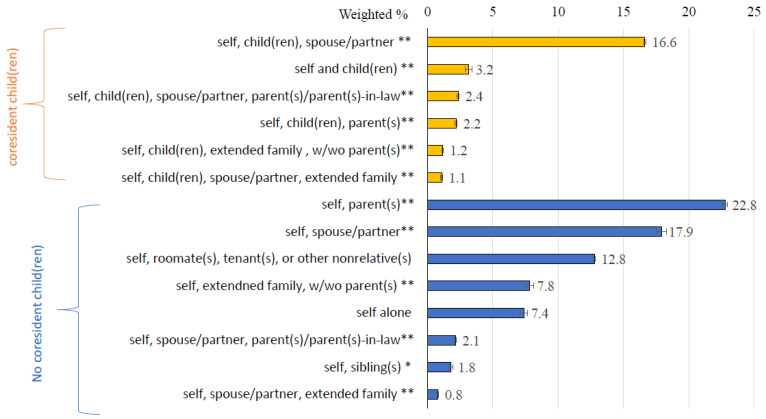
Household compositions among U.S. adults aged 21–29 by the status of the respondent’s coresident child(ren). Data source: The 2016–2019 National Surveys on Drug Use and Health (NSDUH) data. *: May have roommate(s), tenant(s), or other non-relative(s) (RTN). **: May have sibling(s) or RTN. Coresident children = Respondent’s child(ren) [biological/adoptive child(ren) or stepchild(ren)] aged under 18 who resided with the respondent within the same household. w/wo = with or without. Extended family includes grandparent(s), aunt(s), uncle (s), cousin(s), niece(s), nephew(s), or other relative(s) but not parent(s)/parent(s)-in-law. Error bar = standard error.

The U.S. Census Bureau defines a “household” as all the people who occupy a single housing unit, regardless of their relationship to one another [20]. Household composition is determined by the people living together and their relationships with one another. To assess household composition and build a household roster, NSDUH examined each specific relationship of household residents to a sampled respondent up to the 25th oldest person per each sampled household. Due to the importance of residing with their own children on substance use (e.g., opioid misuse [3]), we categorized household composition based on whether a respondent’s child or children, who could be biological, adoptive, or step-children aged under 18, resided with the respondent within the same household (hereafter “*coresident children*”). Household composition was further based on relationships of the young adult respondent with other household residents. To facilitate meaningful analyses, those residing with siblings also may have resided with unrelated individuals. Each of the other types of household composition may have included coresident siblings and/or unrelated individuals, except for household types of those living alone and those living solely with unrelated individuals. See Figure 1 for the 14 mutually exclusive categories examined.

NSDUH collected information about tobacco use, binge alcohol use, and illicit drug use by the respondent during the previous 30 days (hereafter “past-month use”). Tobacco products included cigarettes, smokeless tobacco (such as snuff, dip, chewing tobacco, or snus), cigars, and pipe tobacco. Binge alcohol use was defined as drinking ≥5 drinks for men or ≥4 drinks for women on the same occasion on at least 1 day in the past 30 days. NSDUH defined illicit drug use as any use of marijuana (interchangeable as cannabis, regardless of the legalization status of nonmedical cannabis use in the residing state), cocaine, heroin, inhalants, hallucinogens, and methamphetamine or the misuse of prescription stimulants, tranquilizers, sedatives, and pain relievers. NSDUH defined the misuse of prescription medications as “use in any way not directed by a doctor, including use without a prescription of one’s own; use in greater amounts, more often, or longer than told to take a drug; or use in any other way not directed by a doctor” [18].

The 2016–2019 NSDUH assessed whether adult respondents reported any mental illness (yes/no). Adults were classified as having any mental illness if they had any past-year mental, behavioral, or emotional disorder of sufficient duration to meet DSM-IV criteria [21] (excluding developmental disorders and substance use disorder). NSDUH also asked respondents about sociodemographic characteristics (e.g., age [a continuous variable], sex [male or female], race and ethnicity [non-Hispanic Black, non-Hispanic American Indian or Alaska Native, non-Hispanic Asian or Native Hawaiian/Other Pacific Islander, non-Hispanic individual with more than 1 race, Hispanic, or non-Hispanic White], educational attainment [less than high school education, high school graduate/diploma, some college education, or college graduate], employment status [full-time employment, part-time employment, unemployment, or other], family income [less than $20,000, $20,000–$49,999, $50,000–$74,999, or $75,000 or more], marital status [married, divorced/separated, never married, or unknown], health insurance [private health insurance only, Medicaid only, uninsured, or other], metropolitan statistical area (MSA) status [large MSA, small MSA, or non-MSA]). Details about NSDUH methods and survey questionnaires are available [18].

### 2.3. Statistical Analyses

Among adults aged 21–29 in the U.S., descriptive analyses were conducted to estimate the distribution of household composition by type and national prevalence of past-month substance use, including various combinations of tobacco use (yes/no), binge alcohol use (yes/no), and illicit drug use (yes/no) for a total of eight mutually exclusive outcomes. Next, bivariate multinomial logistic regression [22] was applied to examine these outcomes simultaneously and to estimate the prevalence of past-month substance use by household composition.

Because age, sex, race/ethnicity, educational attainment, employment status, family income, MSA status, and any mental illness are all associated with substance use [1,2,6], it was necessary to control for these factors to better understand the specificity of associations between substance use and household composition. Multivariable multinomial logistic regression [22] was applied to examine the eight outcomes simultaneously after controlling for these potential confounding factors. Each examined variable in our final multivariable logistic regression model had a variance inflation factor smaller than 2.0; thus, multicollinearity was not an issue. Potential interaction effects were also assessed among examined variables and were not found in our final model.

All analyses used SUDAAN software (Release 11.0.3) [23] to account for NSDUH’s complex sample design and sample weights. The NSDUH weighting procedures adjusted for nonresponse through direct adjustments as well as an indirect adjustment via poststratification [24]. For each analysis, *p* < 0.05 (2-tailed) was considered statistically significant.

## 3. Results

### 3.1. Distribution of Household Composition by Type among Young Adults

#### 3.1.1. Study Sample

We examined data on 60,000 civilian noninstitutionalized adult participants aged 21–29 in the 2016–2019 NSDUH. Among them, 49.4% were male; 54.4% were aged 21–25; 21.0% were Hispanic, 2.2% were non-Hispanic adults with ≥1 race, 0.7% were non-Hispanic American Indian or Alaska Native, 13.8% were non-Hispanic Black, 7.5% were non-Hispanic Asian or Native Hawaiian and Other Pacific Islander, and 54.8% were non-Hispanic White.

#### 3.1.2. Types of Household Composition among Young Adults with Coresident Child(ren)

Among adults aged 21–29 with coresident children (Figure 1), the most common type of household composition was those residing with children and a spouse or unmarried partner (hereafter “spouse/partner”) (16.6%, 95% CI = 16.5–16.8%). Moreover, 3.2% (95% CI = 2.7–3.6%) resided with children; 2.4% (95% CI = 2.2–2.5%) resided with children, a spouse/partner, and parents or parents-in-law (hereafter “parents”), and 2.2% (95% CI = 2.0–2.4%) resided with children and parents. Additionally, 1.2% (95% CI = 1.0–1.3%) resided with children, extended family (e.g., grandparents, aunts, uncles, cousins, nieces, nephews, or other relatives) with/without parents; and 1.1% (95% CI = 1.0–1.2%) resided with children, a spouse/partner, and extended family with/without parents.

#### 3.1.3. Types of Household Composition among Young Adults without Coresident Child(ren)

By contrast, among U.S. adults aged 21–29 without coresident children (Figure 1), the common types of household composition included 22.8% (95% CI = 22.2–23.4%) residing with parents without a spouse/partner; 17.9% (95% CI = 17.6–18.3%) residing with a spouse/partner; and 12.8% (95% CI = 12.6–12.9%) solely residing with roommates, tenants, or nonrelatives (hereafter “unrelated individuals”). Moreover, 7.8% (95% CI = 7.5–8.2%) resided with extended family, with/without parents, and 7.4% (95% CI = 6.7–8.1%) lived alone. Additionally, 2.1% (95% CI = 2.0–2.3%) resided with a spouse/partner and parents; 1.8% (95% CI = 1.2–2.4%) resided only with siblings; and 0.8% (95% CI = 0.7–0.9%) resided with a spouse/partner and extended family, with or without parents.

### 3.2. Prevalence of Past-Month Substance Use among Young Adults

**Figure 2:**
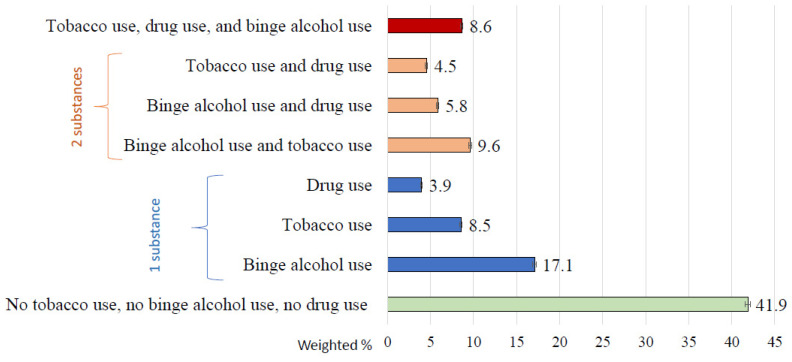
Past-month substance use among adults aged 21–29 in the U.S. Data source: The 2016–2019 National Surveys on Drug Use and Health (NSDUH) data. NSDUH defines drug use as any use of cannabis, heroin, cocaine, inhalants, methamphetamine, or hallucinogens or any misuse of prescription pain relievers, stimulants, sedatives, or tranquilizers. Error bar = standard error.

Among U.S. young adults aged 21–29 in the past month (Figure 2), 41.9% (41.3–42.5%) reported no tobacco, binge alcohol, or drug use; 17.1% (95% CI = 16.7–17.5%) reported binge alcohol use (without tobacco or illicit drug use); 8.5% (8.2–8.9%) reported tobacco use (without binge alcohol or illicit drug use); and 3.9% (95% CI = 3.7–4.2%) reported illicit drug use (without binge alcohol or tobacco use). Moreover, 9.6% (95% CI = 9.3–9.9%) reported binge alcohol and tobacco use (without illicit drug use); 5.8% (95% CI = 5.6–6.1%) reported binge alcohol and illicit drug use (without tobacco use); 4.5% (95% CI = 4.3–4.8%) reported tobacco and illicit drug use (without binge alcohol use); and 8.6% (95% CI = 8.3–8.9%) reported tobacco, binge alcohol, and illicit drug use.

### 3.3. Unadjusted Prevalence of Past-Month Substance Use by Household Composition

The unadjusted prevalence of past-month substance use varied by household composition (Table 1). Among young adults residing with children and a spouse/partner, 50.4% (95% CI = 49.0–51.8%) reported no tobacco, binge alcohol, or illicit drug use, 12.3% (95% CI = 11.4–13.2%) reported binge alcohol use, 14.3% (95% CI = 13.4–15.3%) reported tobacco use, 2.4% (95% CI = 2.0–2.9%) reported binge alcohol use and drug use, and 4.3% (95% CI = 3.9–4.9%) reported tobacco, binge alcohol, and illicit drug use.

By contrast, among young adults residing solely with unrelated individuals, 29.6% (95% CI = 27.9–31.4%) reported no tobacco, binge alcohol, or illicit drug use, 22.1% (95% CI = 20.8–23.5%) reported binge alcohol use, 5.6% (95% CI = 4.8–6.4%) reported tobacco use, 10.9% (95% CI = 9.9–12.0%) reported binge alcohol use and drug use, and 14.1% (95% CI = 12.9–15.3%) reported tobacco, binge alcohol, and illicit drug use. Almost all these estimates were significantly higher among young adults residing solely with unrelated individuals than those residing with children and a spouse/partner.

### 3.4. Adjusted Prevalence of Past-Month Substance Use by Household Composition

#### 3.4.1. Adjusted Prevalence of Binge Alcohol Use, Illicit Drug Use, and Tobacco Use by Household Composition

Even after controlling for potential confounding factors, multivariable multinomial logistic regression results showed that the adjusted prevalence of past-month substance use varied by household composition among young adults (Table 2 and Table 3). Binge alcohol use prevalence was higher among those residing with a spouse/partner, those living alone, those residing solely with unrelated individuals, and those residing with parents than those with coresident children and a spouse/partner (adjusted prevalence [AP] = 16.3–23.7% vs. 13.5%; adjusted prevalence ratios [APRs] = 1.2–1.8, 95% CIs = 1.1–1.9). Similarly, illicit drug use prevalence was higher among those residing with a spouse/partner, those living alone, those residing solely with unrelated individuals, those residing with parents, and those residing with extended family with/without parents than those with coresident children and a spouse/partner (AP = 4.0–4.7% vs. 2.9%; APRs = 1.4–1.6, 95% CIs = 1.1–2.1). However, tobacco use prevalence was lower among those residing with a spouse/partner, those living alone, those residing only with unrelated individuals, those residing with siblings, those residing with parents, and those residing with extended family with/without parents than those with coresident children and a spouse/partner (AP = 5.3–8.4% vs. 12.2%; APRs = 0.4–0.7, 95% CIs = 0.4–0.8).

#### 3.4.2. Adjusted Prevalence of Binge Alcohol and Tobacco Use, Binge Alcohol and Drug Use, and Tobacco and Drug Use by Household Composition

Adjusted prevalence of reporting both binge alcohol and tobacco use was higher among those residing with children, those residing with children and parents, and those living alone than those with coresident children and a spouse/partner (AP = 12.1–15.1% vs. 9.3%; APRs = 1.3–1.6, 95% CIs = 1.1–1.9) (Table 2 and Table 3). However, reporting both binge alcohol and tobacco use was lower among those residing with parents than those with coresident children and a spouse/partner (AP = 8.2% vs. 9.3%; APR = 0.9, 95% CI = 0.8–1.0).

Adjusted prevalence of reporting both binge alcohol and drug use was higher among almost all examined types of household composition without children than those with children and a spouse/partner (AP = 4.8–10.9% vs. 3.1%; APRs = 1.6–3.5, 95% CIs = 1.3–4.4) (Table 2 and Table 3). The adjusted prevalence of reporting both tobacco and drug use was lower among those residing only with unrelated individuals than those with children and a spouse/partner (AP = 3.4% vs. 4.1%; APR = 0.8, 95% CI = 0.6–1.0). However, the adjusted prevalence of reporting both tobacco and drug use was higher among most other examined types of household composition without children than those with child(ren) and a spouse/partner (AP = 5.0–9.6% vs. 4.1%; APRs = 1.2–2.3, 95% CIs = 1.0–3.4).

#### 3.4.3. Adjusted Prevalence of Tobacco, Drug, and Binge Alcohol Use by Household Composition

The highest adjusted prevalence of reporting all three categories (tobacco, drug, and binge alcohol) was 13.8% (95% CI = 12.5–15.1%) among those residing solely with unrelated individuals (Table 2). Adjusted prevalence of reporting all three categories was higher among all examined household compositions without children than those with children and a spouse/partner (AP = 7.7–13.8% vs. 4.9%; APRs = 1.6–2.8, 95% CIs = 1.2–3.3) (Table 2 and Table 3). In addition, the adjusted prevalence of reporting all three categories was higher among those residing with children and those residing with children and parents than those with coresident children and a spouse/partner (AP = 9.1–9.3% vs. 4.9%; APRs = 1.9–1.9, 95% CIs = 1.5–2.4).

**Table 2:** Model-adjusted prevalence of past-month substance use among U.S. adults aged 21–29 by household composition, weighted % (95% CI).

House Composition: Self Plus	No Substance Use	Binge Alcohol Use	Tobacco Use	Drug Use	Binge Alcohol Use and Tobacco Use	Binge Alcohol Use and Drug Use	Tobacco Use and Drug Use	Tobacco Use, Drug Use, and Binge Alcohol Use
n = 60,000 ^C^								
**With coresident child(ren)**								
child(ren), spouse/partner ^B^ +	49.9 (48.4–51.3)	13.5 (12.6–14.6)	12.2 (11.4–13.1)	2.9 (2.4–3.5)	9.3 (8.7–10.1)	3.1 (2.6–3.7)	4.1 (3.6–4.7)	4.9 (4.4–5.5)
child(ren) ^B^	**38.5** **(36.0–41.0)**	14.4 (12.5–16.6)	12.4 (10.8–14.3)	3.3 (2.4–4.5)	**15.1** **(13.1–17.3)**	3.4 (2.5–4.8)	3.7 (2.9–4.9)	**9.1** **(7.5–11.1)**
child(ren), spouse/partner, parent(s)/parent(s)-in-law ^B^	**45.0** **(41.6–48.4)**	13.9 (11.3–17.1)	12.8 (10.7–15.1)	4.1 (2.8–5.9)	9.3 (7.6–11.4)	3.7 (2.4–5.6)	5.1 (3.7–7.0)	6.2 (4.7–8.2)
child(ren), parent(s) ^B^	**38.2** **(35.0–41.5)**	13.5 (11.3–16.0)	14.3 (12.0–16.9)	2.6 (1.7–4.0)	**14.1** **(11.4–17.2)**	2.9 (2.0–4.2)	5.2 (3.9–7.0)	**9.3** **(7.3–11.6)**
child(ren), extended family, w/wo parent(s) ^B^	**40.3** **(35.5–45.2)**	17.3 (13.0–22.5)	13.9 (10.9–17.6)	3.7 (2.4–5.7)	9.7 (7.1–13.0)	2.3 (1.3–4.3)	6.5 (4.1–10.2)	6.4 (4.4–9.1)
child(ren), extended family, spouse/partner, w/wo parent(s)/parent(s)-in-law ^B^	**41.6** **(36.2–47.2)**	14.5 (10.9–19.1)	13.1 (10.1–16.7)	4.5 (2.7–7.5)	9.1 (6.2–13.3)	4.7 (2.5–8.7)	6.0 (4.1–8.9)	6.4 (4.4–9.1)
**Without coresident child(ren)**								
parent(s) ^B^	**46.5** **(45.3–47.7)**	**16.3** **(15.5–17.3)**	**6.8** **(6.2–7.5)**	**4.1** **(3.6–4.6)**	**8.2** **(7.6–8.9)**	**4.8** **(4.4–5.3)**	**5.1** **(4.5–5.7)**	**8.2** **(7.6–8.9)**
spouse/partner ^B^	**41.4** **(40.0–42.7)**	**18.3** **(17.5–19.3)**	**7.3** **(6.6–8.0)**	**4.7** **(4.2–5.4)**	8.8 (8.1–9.5)	**6.4** **(5.8–7.1)**	**5.0** **(4.4–5.6)**	**8.1** **(7.4–8.8)**
roommate(s), tenant(s), or other nonrelative(s)	**28.6** **(27.0–30.3)**	**23.7** **(22.1–25.3)**	**5.3** **(4.6–6.2)**	**4.3** **(3.6–5.1)**	10.4 (9.3–11.6)	**10.9** **(9.8–12.1)**	**3.1** **(2.7–3.7)**	**13.8** **(12.5–15.1)**
extended family, w/wo parent(s) ^B^	**42.7** **(40.7–44.8)**	14.1 (12.7–15.6)	**8.4** **(7.3–9.6)**	**4.1** **(3.3–5.0)**	10.2 (9.1–11.5)	**5.4** **(4.5–6.3)**	**5.6** **(4.8–6.5)**	**9.6** **(8.5–10.8)**
self alone	**38.1** **(36.1–40.2)**	**20.5** **(19.0–22.2)**	**5.6** **(4.9–6.4)**	**4.0** **(3.3–4.8)**	**12.1** **(10.8–13.5)**	**7.3** **(6.4–8.4)**	3.4 (2.8–4.1)	**9.1** **(8.1–10.2)**
parent(s)/parent(s)-in-law, spouse/partner ^B^	**39.9** **(36.0–43.9)**	14.9 (12.4–17.8)	12.0 (9.5–15.0)	4.0 (2.7–5.8)	10.1 (8.0–12.6)	3.8 (2.7–5.5)	**7.7** **(5.7–10.3)**	**7.7** **(5.9–10.2)**
sibling(s) ^A^	**41.7** **(37.8–45.7)**	15.6 (13.0–18.6)	**7.3** **(5.6–9.5)**	2.5 (1.6–4.0)	10.0 (8.0–12.5)	**7.4** **(5.7–9.5)**	3.8 (2.7–5.4)	**11.6** **(9.1–14.7)**
extended family, spouse/partner, w/wo parent(s)/parent(s)-in-law ^B^	**33.0** **(27.9–38.4)**	15.8 (11.9–20.7)	11.4 (8.1–15.7)	4.5 (2.6–7.8)	9.7 (6.7–13.8)	**7.5** **(4.7–11.8)**	**9.6** **(6.8–13.3)**	**8.6** **(6.1–12.1)**

Data source: The 2016–2019 National Surveys on Drug Use and Health (NSDUH) data. Drug use = Any use of cannabis, heroin, cocaine, inhalants, methamphetamine, or hallucinogens or misuse of prescription pain relievers, stimulants, sedatives, or tranquilizers. Coresident children = Respondent’s child(ren) [biological/adoptive child(ren) or stepchild(ren)] aged under 18 who resided with the respondent within the same household. Spouse/Partner = a spouse or unmarried partner. w/wo = with or without. Extended family includes grandparent(s), aunt, uncle, cousin(s), niece(s), nephew(s), or other relatives but not parent(s)/parent(s)-in-law. ^A^: May also have a roommate(s), tenant (s), or other nonrelative(s) (RTN). ^B^: May also have RTN and may also have siblings. ^C^: After dropping 135 (0.22%) of other miscellaneous categories; for restricted NSDUH data, overall and subgroup sample sizes were rounded to the nearest 100 to reduce data disclosure risk. Each estimate is adjusted for age, sex, race/ethnicity, education, family income, employment status, any mental illness, and metropolitan statistical area. +: Reference group. Bold estimates are statistically significantly different from the reference group within each column (*p* < 0.05).

**Table 3:** Adjusted prevalence ratio (95% CI) for associations between household composition and past-month substance use in U.S. adults aged 21–29.

House Composition: Self Plus	No Substance Use	Binge Alcohol Use	Tobacco Use	Drug Use	Binge Alcohol Use and Tobacco Use	Binge Alcohol Use and Drug Use	Tobacco Use and Drug Use	Tobacco Use, Drug Use, and Binge Alcohol Use
n = 60,000 ^C^								
**With coresident child(ren)**								
child(ren), spouse/partner ^B^ +	1.0 (Reference)	1.0 (Reference)	1.0 (Reference)	1.0 (Reference)	1.0 (Reference)	1.0 (Reference)	1.0 (Reference)	1.0 (Reference)
child(ren) ^B^	**0.8** **(0.7–0.8)**	1.1 (0.9–1.3)	1.0 (0.9–1.2)	1.1 (0.8–1.6)	**1.6** **(1.4–1.9)**	1.1 (0.8–1.6)	0.9 (0.7–1.2)	**1.9** **(1.5–2.3)**
child(ren), spouse/partner, parent(s)/parent(s)-in-law ^B^	**0.9** **(0.8–1.0)**	1.0 (0.8–1.3)	1.0 (0.9–1.3)	1.4 (0.9–2.1)	1.0 (0.8–1.2)	1.2 (0.7–1.9)	1.2 (0.9–1.7)	1.3 (0.9–1.7)
child(ren), parent(s) ^B^	**0.8** **(0.7–0.8)**	1.0 (0.8–1.2)	1.2 (1.0–1.4)	0.9 (0.6–1.4)	**1.5** **(1.2–1.9)**	0.9 (0.6–1.4)	1.3 (0.9–1.7)	**1.9** **(1.5–2.4)**
child(ren), extended family, w/wo parent(s) ^B^	**0.8** **(0.7–0.9)**	1.3 (1.0–1.7)	1.1 (0.9–1.5)	1.3 (0.8–2.0)	1.0 (0.8–1.4)	0.8 (0.4–1.4)	1.6 (1.0–2.5)	1.3 (0.9–1.9)
child(ren), extended family, spouse/partner, w/wo parent(s)/parent(s)-in-law ^B^	**0.8** **(0.7–1.0)**	1.1 (0.8–1.4)	1.1 (0.8–1.4)	1.6 (0.9–2.6)	1.0 (0.7–1.5)	1.5 (0.8–2.9)	1.5 (1.0–2.2)	1.3 (0.9–1.9)
**Without coresident child(ren)**								
parent(s) ^B^	**0.9** **(0.9–1.0)**	**1.2** **(1.1–1.3)**	**0.6** **(0.5–0.6)**	**1.4** **(1.1–1.7)**	**0.9** **(0.8–1.0)**	**1.6** **(1.3–1.9)**	**1.2** **(1.0–1.5)**	**1.7** **(1.4–1.9)**
spouse/partner ^B^	**0.8** **(0.8–0.9)**	**1.4** **(1.2–1.5)**	**0.6** **(0.5–0.7)**	**1.6** **(1.3–2.1)**	0.9 (0.9–1.0)	**2.1** **(1.7–2.5)**	**1.2** **(1.0–1.4)**	**1.6** **(1.4–1.9)**
roommate(s), tenant(s), or other nonrelative(s)	**0.6** **(0.5–0.6)**	**1.8** **(1.6–1.9)**	**0.4** **(0.4–0.5)**	**1.5** **(1.1–1.9)**	1.1 (1.0–1.3)	**3.5** **(2.8–4.4)**	**0.8** **(0.6–0.9)**	**2.8** **(2.4–3.3)**
extended family, w/wo parent(s) ^B^	**0.9** **(0.8–0.9)**	1.0 (0.9–1.2)	**0.7** **(0.6–0.8)**	**1.4** **(1.1–1.8)**	1.1 (0.9–1.3)	**1.7** **(1.4–2.2)**	**1.4** **(1.1–1.7)**	**1.9** **(1.7–2.3)**
self alone	**0.8** **(0.7–0.8)**	**1.5** **(1.4–1.7)**	**0.5** **(0.4–0.5)**	**1.4** **(1.1–1.8)**	**1.3** **(1.1–1.5)**	**2.4** **(1.9– 3.0)**	0.8 (0.6–1.0)	**1.8** **(1.6–2.2)**
parent(s)/parent(s)-in-law, spouse/partner ^B^	**0.8** **(0.7–0.9)**	1.1 (0.9–1.3)	1.0 (0.8–1.2)	1.4 (0.9–2.1)	1.1 (0.9–1.4)	1.2 (0.8–1.9)	**1.9** **(1.4–2.6)**	**1.6** **(1.2–2.1)**
sibling(s) ^A^	**0.8** **(0.8–0.9)**	1.2 (1.0–1.4)	**0.6** **(0.5–0.8)**	0.9 (0.5–1.4)	1.1 (0.9–1.4)	**2.4** **(1.7–3.3)**	0.9 (0.7–1.3)	**2.4** **(1.8–3.1)**
extended family, spouse/partner, w/wo parent(s)/parent(s)-in-law ^B^	**0.7** **(0.6–0.8)**	1.2 (0.9–1.6)	0.9 (0.7–1.3)	1.6 (0.9–2.8)	1.0 (0.7–1.5)	**2.4** **(1.5–4.0)**	**2.3** **(1.6–3.4)**	**1.8** **(1.2–2.5)**

Data source: The 2016–2019 National Surveys on Drug Use and Health (NSDUH) data. Drug use = Any use of cannabis, heroin, cocaine, inhalants, methamphetamine, or hallucinogens or misuse of prescription pain relievers, stimulants, sedatives, or tranquilizers. No substance use = No tobacco use, drug use, or binge alcohol use. Coresident children = A respondent’s child(ren) [biological/adoptive child(ren) or stepchild(ren)] aged under 18 who resided with the respondent within the same household. Spouse/Partner = A spouse or unmarried partner. w/wo = with or without. Extended family includes grandparent(s), aunt, uncle, cousin(s), niece(s), nephew(s), or other relatives but not parent(s)/parent(s)-in-law. ^A^: May also have roommate(s), tenant (s), or other nonrelative(s) (RTN). ^B^: May also have RTN and may also have siblings. ^C^: After dropping 135 (0.22%) of other miscellaneous categories; for restricted NSDUH data, overall and subgroup sample sizes were rounded to the nearest 100 to reduce data disclosure risk. Each estimate is adjusted for age, sex, race/ethnicity, education, family income, employment status, any mental illness, and metropolitan statistical area. +: Reference group. Bold estimates are statistically significantly different from the reference group within each column (*p* < 0.05).

## 4. Discussion

We find that young adults in the U.S. live in highly diverse households. In particular, our results show how specific patterns of binge alcohol, tobacco, and illicit drug use vary by household composition. To the best of our knowledge, this is the first study detailing relationships between 14 mutually exclusive types of household composition and 8 mutually exclusive substance use patterns among young adults in the U.S.

Overall, our results extend existing findings on related topics. For example, researchers have reported that marriage and parenthood are associated with lower risks for opioid misuse [7]. Moreover, earlier research has shown that marriage or cohabitation is associated with lower risks for binge alcohol use among women [2] and for alcohol, tobacco, and cannabis use among men [25]. Consistently, a longitudinal study [26] found that substance use is associated with reduced rates of marriage and parenthood among young adults in the U.S. In our study among young adults in the U.S., we found that those residing solely with unrelated individuals had a higher prevalence of most examined substance use patterns for tobacco, binge alcohol, and drug use. By contrast, we also found that living with a spouse/partner and one’s own child(ren) was associated with a lower prevalence of most examined substance use patterns. Consistent with these findings, a longitudinal study of young Swiss men who lived with their family at baseline and were followed for up to 15 months found that moving out to live with peers and a lack of parental monitoring were associated with substance use initiation [27]. Taken together, these results suggest that substance use prevention and intervention strategies may benefit from targeting households, rather than just individuals, to best meet the needs of those who live in diverse household situations.

For the prevalence of binge alcohol use, illicit drug use, binge alcohol and drug use, and tobacco and drug use, young adults with coresident children and a spouse/partner did not differ from any other household compositions with coresident children but had a lower prevalence than most household compositions without coresident children. These results suggest that coresiding with one’s own children within the same household may be a protective factor for these four substance use patterns. Consistently, researchers have suggested that the low prevalence of parenthood among young people who use substances may result from concerns about the potential negative effects of substance use behaviors on their children [26]. However, the percentage of households with coresident child(ren) in the U.S. has been declining and the downward trend is projected to continue [8]. More young adults now delay getting a job, marrying, and having children compared to their parents [5]. Our results suggest that these demographic shifts may indicate the greater vulnerability of young adults of Generation Z and future generations to substance use compared to earlier generations. If not mitigated through prevention strategies, these demographic changes may potentially lead to increases in substance use, substance use disorders, overdose, other related comorbidities, and premature mortality.

Moreover, compared to those residing with child(ren) and a spouse/partner, the prevalence of binge alcohol and tobacco use and the prevalence of binge alcohol, tobacco, and drug use were higher among young adults residing with child(ren) as well as among young adults residing with their child(ren) and parent(s). These results suggest that the presence of a spouse/partner may serve as a protective factor for these two patterns of substance use. In prior research, after examining male–male adult twin pairs from a state population-based registry in the U.S. and by using co-twin and within-person analyses, researchers have found that married men use less alcohol, tobacco, and cannabis than divorced/separated or single men, and these reductions begin before marriage [25]. However, the share of U.S. children residing with a single parent has been increasing for decades [28,29]. Moreover, even for young adults with a coresident child(ren) and a spouse/partner, half reported binge alcohol, tobacco, or illicit drug use in the past month. Furthermore, researchers have reported that parental substance use (e.g., marijuana use, nonmedical use of prescription opioids, and cigarette smoking) is associated with increased risk of substance use among coresident offspring [14,16]. Taken together, these results suggest that evidence-based substance use prevention intervention strategies may be increasingly warranted to meet the needs of evolving household compositions.

For the prevalence of tobacco use, we find that young adults residing with their child(ren) and a spouse/partner-only do not differ from young adults with other types of household compositions with coresident child(ren). However, young adults residing with their child(ren) and a spouse/partner only have a higher prevalence of tobacco use than most types of household composition without coresident child(ren). A recent study reported that parenting stress is associated with greater tobacco use [30]. Another recent study highlights the complexity of socioeconomic disparities in smoking prevalence in Sweden [31]. In addition to parenting stress, young couples aged 21–29 residing with children who may not have advanced education, professional occupations, and high family income may be under financial stress. Importantly, parental smoking is associated with the substance use of coresident adolescent children [16]. Moreover, secondhand smoke exposure can cause respiratory infections and asthma among children, sudden infant death syndrome among infants, and cardiovascular diseases, lung cancer, and premature death among adults [32]. Thus, our results suggest that targeted tobacco control and other substance use prevention and intervention efforts along with broader strategies to alleviate economic insecurity and related stressors may be particularly helpful for young adults residing with their children.

Residing solely with unrelated individual(s) is another common type of household composition among young adults in the U.S. Across all examined household composition subtypes, those residing solely with unrelated individual(s) had the highest prevalence of past-month substance use. Similar findings have been reported in young Swiss men based on a longitudinal study [27]. This type of household composition appears vulnerable to various patterns of substance use and may benefit from targeted efforts for substance use prevention, treatment, and harm reduction that consider their unique social and structural networks.

This study has several limitations. First, NSDUH is a self-report survey and is subject to recall and social-desirability biases. For example, young adults with coresident children may underreport their drug use due to fears of child protective services. Second, for respondents with coresident child(ren), we did not examine the number and age of the children. Third, the cross-sectional nature of NSDUH data precludes drawing causal inferences between household structure and substance use. For example, factors not assessed by the NSDUH (e.g., loneliness [33], social connectedness [34], stress [35], cultural norms [36,37], perceived social support [38], and neighborhood disadvantage [39,40]) may confer a shared likelihood between marriage and low substance use and between having children and tobacco use. An Australian study reported sex differences in substance use disorder (i.e., alcohol use disorder) by marital status and parenthood among midlife adults [41]. A Swedish population-based study found that among married people, having coresident children was associated with a reduced risk for alcohol use disorder, which was stronger in mothers than fathers and with younger children than older children [42]. Future longitudinal research assessing these factors is needed to improve understanding of the critical contributions of social determinants of patterns of substance use and substance use disorders and cultural practices and to clarify their roles in evidence-based interventions meeting the needs of all household compositions. Notably, longitudinal research is needed to examine specific substance use initiation and use patterns in the context of each household composition according to various personal and sociodemographic characteristics (e.g., age, sex, race/ethnicity, educational attainment, employment status, family income, and health status), detailed social network, social support, social connectedness, neighborhood factors, and underlying mental disorders. Additionally, studies are needed to assess household composition and cannabis use and use disorder among male and female college-attending and non-college-attending young adults.

Despite these limitations, our study shows that U.S. young adults live in highly diverse households and offers insights into how 8 mutually exclusive patterns of binge alcohol, tobacco, and illicit drug use vary by 14 mutually exclusive types of household composition. Our results may help inform substance use prevention and intervention strategies directly focusing on the household level. Among U.S. young adults, the presence of a young adult’s own children and a spouse/partner is associated with lower risk for most examined patterns of substance use. Across all examined household composition subtypes, those residing only with unrelated individuals have the highest prevalence of substance use. As household compositions continue to diversify, tailored evidence-based substance use prevention and intervention strategies may be warranted to meet the needs of diverse household compositions.

## Figures and Tables

**Table 1:** Prevalence of past-month substance use among U.S. adults aged 21–29 by household composition, weighted % (95% CI).

House Composition: Self Plus	No Substance Use	Binge Alcohol Use	Tobacco Use	Drug Use	Binge Alcohol Use and Tobacco Use	Binge Alcohol Use and Drug Use	Tobacco Use and Drug Use	Tobacco Use, Drug Use, and Binge Alcohol Use
n = 60,000 ^C^								
**With coresident child(ren)**								
child(ren), spouse/partner ^B^ +	50.4 (49.0–51.8)	12.3 (11.4– 13.2)	14.3 (13.4–15.3)	2.5 (2.1–2.9)	9.7 (9.0–10.4)	2.4 (2.0–2.9)	4.1 (3.6–4.7)	4.4 (3.9–4.9)
child(ren) ^B^	**44.3** **(41.7–47.0)**	12.3 (10.6–14.1)	13.9 (12.2–15.9)	3.3 (2.4–4.5)	11.1 (9.6–12.7)	2.7 (1.9–3.7)	4.9 (3.9–6.2)	**7.6** **(6.2–9.2)**
child(ren), spouse/partner, parent(s)/parent(s)-in-law ^B^	46.2 (42.5–49.8)	13.5 (10.8–16.6)	13.1 (10.9–15.6)	3.7 (2.5–5.4)	9.7 (7.9–12.0)	3.3 (2.1–5.0)	4.7 (3.4–6.5)	5.9 (4.4–7.8)
child(ren), parent(s) ^B^	**43.2** **(39.6–46.9)**	13.3 (11.2–15.9)	13.7 (11.5–16.3)	2.7 (1.7–4.2)	11.1 (8.9–13.7)	2.7 (1.9–3.9)	5.3 (4.0–7.0)	**8.0** **(6.3–10.1)**
child(ren), extended family, w/wo parent(s) ^B^	**44.4** **(39.5–49.5)**	15.7 (11.9–20.4)	14.2 (11.1–18.0)	3.7 (2.4–5.7)	7.4 (5.5–10.0)	2.0 (1.1–3.7)	**7.0** **(4.5–10.7)**	5.5 (3.8–7.9)
child(ren), extended family, spouse/partner, w/wo parent(s)/parent(s)-in-law ^B^	44.0 (38.4–49.8)	13.2 (9.8–17.6)	13.9 (10.6–17.9)	**4.2** **(2.5–6.9)**	8.8 (6.0–12.7)	4.0 (2.2–7.4)	6.0 (4.0–9.0)	5.9 (4.1–8.4)
**Without coresident child(ren)**								
parent(s) ^B^	**45.3** **(44.2–46.4)**	**17.5** **(16.7–18.4)**	**5.7** **(5.2–6.2)**	**4.3** **(3.9–4.8)**	**8.3** **(7.7–8.9)**	**5.6** **(5.2–6.1)**	4.5 (4.0–5.0)	**8.8** **(8.2–9.5)**
spouse/partner ^B^	**39.6** **(38.3–40.9)**	**20.9** **(19.9–21.9)**	**6.7** **(6.1–7.4)**	**4.5** **(3.9–5.1)**	9.3 (8.6–10.0)	**7.0** **(6.4–7.8)**	4.2 (3.7–4.7)	**7.9** **(7.2–8.7)**
roommate(s), tenant(s), or other nonrelative(s)	**29.6** **(27.9–31.4)**	**22.1** **(20.8–23.5)**	**5.6** **(4.8–6.4)**	**4.5** **(3.8–5.2)**	9.7 (8.7–10.8)	**10.9** **(9.9–12.0)**	3.6 (3.0–4.2)	**14.1** **(12.9–15.3)**
extended family, w/wo parent(s) ^B^	**42.0** **(40.0–44.1)**	12.9 (11.7–14.3)	**8.3** **(7.3–9.5)**	**4.5** **(3.7–5.5)**	10.1 (9.0–11.4)	**5.3** **(4.4–6.2)**	**6.4** **(5.5–7.3)**	**10.5** **(9.4–11.7)**
self alone	**37.5** **(35.6–39.5)**	**19.5** **(18.1–21.1)**	**6.2** **(5.4–7.1)**	**4.2** **(3.5–5.0)**	**12.3** **(11.1–13.7)**	**7.0** **(6.2–8.0)**	3.9 (3.2–4.8)	**9.3** **(8.3–10.4)**
parent(s)/parent(s)-in-law, spouse/partner ^B^	**39.0** **(35.2–43.0)**	**15.4** **(12.7–18.5)**	11.4 (9.0–14.2)	**3.9** **(2.6–5.6)**	10.7 (8.6–13.4)	**4.1** **(2.9–5.9)**	**7.2** **(5.1–9.9)**	**8.4** **(6.4–10.9)**
sibling(s) ^A^	**40.1** **(36.0–44.3)**	14.4 (12.0–17.2)	**7.7** **(5.9–10.0)**	2.8 (1.8–4.4)	10.5 (8.4–13.1)	**7.2** **(5.5–9.3)**	4.4 (3.2–6.2)	**12.8** **(10.1–16.2)**
extended family, spouse/partner, w/wo parent(s)/parent(s)-in-law ^B^	**32.9** **(27.4–38.9)**	16.0 (11.9–21.2)	11.0 (7.7–15.3)	4.4 (2.5–7.6)	10.0 (7.0–14.2)	**7.5** **(4.6–12.0)**	**9.3** **(6.5–13.2)**	**8.9** **(6.3–12.4)**

Data source: The 2016–2019 National Surveys on Drug Use and Health (NSDUH) data. Drug use = Any use of cannabis, heroin, cocaine, inhalants, methamphetamine, or hallucinogens or misuse of prescription pain relievers, stimulants, sedatives, or tranquilizers. No substance use = No tobacco use, drug use, or binge alcohol use. Coresident children = A respondent’s child(ren) [biological/adoptive child(ren) or stepchild(ren)] aged under 18 who resided with the respondent within the same household. Spouse/Partner = a spouse or unmarried partner. w/wo = with or without. Extended family includes grandparent(s), aunt, uncle, cousin(s), niece(s), nephew(s), or other relatives but not parent(s)/parent(s)-in-law. +: Reference group. Bold estimates are statistically significantly different from the reference group within each column (*p* < 0.05). ^A^: May also have a roommate(s), tenant(s), or other nonrelative(s) (RTN). ^B^: May also have RTN and may also have siblings. ^C^: After dropping 135 (0.22%) of other miscellaneous categories; for restricted NSDUH data, overall and subgroup sample sizes were rounded to the nearest 100 to reduce data disclosure risk.

## Data Availability

Due to potential data disclosure risk, detailed household composition variables are only available in restricted National Surveys on Drug Use and Health (NSDUH) data files and are unavailable in NSDUH public use files. For more information regarding data access, please visit: https://www.samhsa.gov/data/data-we-collect/samhsa-rdc.
